# Bipolar Electrodes for Next‐Generation Rechargeable Batteries

**DOI:** 10.1002/advs.202001207

**Published:** 2020-07-16

**Authors:** Tiefeng Liu, Yifei Yuan, Xinyong Tao, Zhan Lin, Jun Lu

**Affiliations:** ^1^ College of Materials Science and Engineering Zhejiang University of Technology Hangzhou 310014 China; ^2^ Chemical Sciences and Engineering Division Argonne National Laboratory Lemont IL 60439 USA; ^3^ College of Chemical and Biological Engineering Zhejiang University Hangzhou 310027 China

**Keywords:** bipolar electrodes, electrode stacks, high power, high voltage, rechargeable batteries

## Abstract

The development of advanced rechargeable batteries provides a great opportunity for basic and applied researchers to collectively overcome challenging scientific and technological barriers that directly address a critical need for energy storage. In addition to novel battery chemistries often scientifically reviewed, advanced battery structures via technological innovations that boost battery performance are also worthy of attention. In this context, bipolar electrodes (BEs) are capable of improving the specific power, simplifying cell components, and reducing manufacturing costs for rechargeable batteries. By focusing on the fundamentals and applications of BEs in rechargeable batteries, the rational utilization of BEs from an academic perspective is considered. The progress and challenges of BEs are discussed and summarized in detail. Key techniques and materials for enabling BEs are highlighted and an outlook for the future directions of BEs that involve emerging concepts, such as wearable devices, all‐solid‐state batteries, fast spraying fabrication, and recyclable secondary batteries, is also presented.

## Graphical abstract

Bipolar electrodes (BEs) offer numerous advantages of simplifying battery components, boosting specific power, increasing specific energy, and lowering manufacturing cost to target next‐generation rechargeable batteries. The infiltration of BEs into emerging applications including wearable technologies, solid‐state electrolytes, 3D spraying fabrications, and recyclable batteries provides a great prospect.

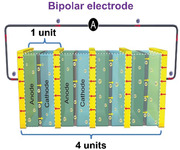

## 1 Introduction

In 1800, the Italian physicist Alessandro Volta invented voltaic piles (cells) that consisted of copper and zinc disks for the electrodes and a layer of cloth or cardboard soaked in brine for a separator, which successfully produced a continuous and stable current.^[^ ^1^ ^]^ This apparatus is the prototype for a rechargeable battery based on reversible chemical reactions to store and release electricity.^[^ ^2^ ^]^ Since then, rechargeable batteries have undergone countless improvements in electrode materials and cell configurations for higher specific energy, higher specific power, longer lifetime, better safety, and lower cost.^[^ ^3^, ^4^ ^]^ Up to now, such challenges have been progressively addressed by intensive scientific research into the development of novel battery chemistries,^[^ ^5^, ^6^, ^7^ ^]^ such as high‐capacity electroactive materials, electrolytes, and binders. In contrast, the role of a novel battery structure in boosting battery performance has been often overlooked in academia, and less effort has been devoted to its exploration.

**Figure 1:**
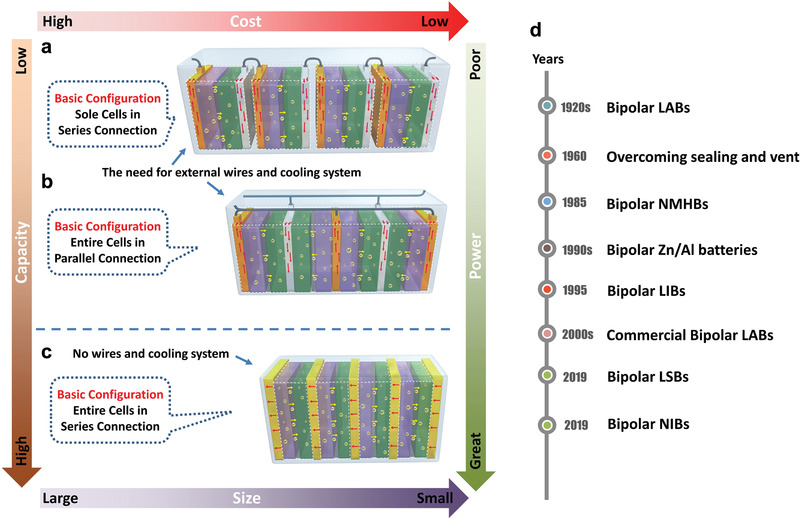
Schematic illustration of the stack configuration in rechargeable batteries: a) SEs, b) MEs, and c) BEs. The direction and amount of red arrow represent the discharging current. d) The timeline of these key works and development in the history of BEs.

Actually, the optimization of the battery structure, though belonging to technological innovations, has played a crucial role in improving battery performance in the industrial sector.^[^ ^8^ ^]^ Typically, the shift in cell configurations from single electrodes (SEs) to monopolar electrodes (MEs) has facilitated less use of the substrate (often called as current collector), as shown in **Figure** **1**a,b. Meanwhile, all the electrodes use the shared electrolyte, thus decreasing the amount of electrolyte in the entire battery case. More active materials can be incorporated into a limited battery case for higher capacity storage.^[^ ^9^ ^]^ Consequently, capacity, cost, and size of the rechargeable batteries are improved simultaneously. For example, the first commercial lithium‐ion battery (LIB) was assembled by LiCoO_2_ cathode and graphite anode. Despite of using identical materials as before, the engineers have successfully increased the energy density of such a LIB to 200 Wh kg^−1^ from an initial 80 Wh kg^−1^ through substantial optimizations in cell configurations.^[^ ^5^ ^]^ As such, the interests in developing advanced cell configurations for better rechargeable batteries should be aroused.

There is a distinctive stack configuration of rechargeable batteries, referred to as bipolar electrodes (BEs), that ultimately simplifies the components of rechargeable batteries.^[^ ^10^ ^]^ A schematic illustration of BEs is displayed in Figure 1c. The cathode and anode slurries are separately coated on both sides of the substrate. Electron transfer between the cathode and the anode occurs through this substrate without an external connector, resulting in a short electron transfer for lower ohmic resistance and a homogeneous current distribution for lower heat production. As a result, BEs render two fascinating advantages of power sources, compared with SEs and MEs.^[^ ^11^, ^12^ ^]^ First, a simple and compact design for a high specific energy. Second, a high‐voltage and current output for a high specific power. Thus such a cell configuration allows both high capacity and power (Figure 1), like a combination of batteries and capacitors. Hitherto, BEs have successfully applied in lead‐acid batteries (LABs) and nickel metal hydride batteries (NMHBs) and are making in‐roads into LIBs and post‐LIBs battery technologies.

This review aims to place the development of BEs in a historical context and brings BEs into the perspective of academic research. We begin by briefly introducing the fundamentals of BEs. Practical applications for BEs in different rechargeable batteries are subsequently discussed. Finally, challenges and opportunities in BEs are summarized. Key techniques and materials for enabling BEs are also highlighted. In particular, the future directions of BEs employed in LIBs and post‐LIBs are analyzed. The infiltration of BEs into emerging technologies, including wearable technology applications, all‐solid‐state batteries, fast spraying fabrication, and recyclable rechargeable batteries, also provides great prospects.

## 2 Fundamentals of the Bipolar Electrodes

Historically, Kaptiza et al. first developed a BE employed in LABs in the early 1920s.^[^ ^13^ ^]^ However, the issues of sealing and serious substrate corrosion in Kaptiza‐type bipolar LABs cause the battery failure. Until 1960s, the work by Biddick and Nelson has confirmed the necessity of seals and vents in bipolar LABs.^[^ ^14^ ^]^ Since 1960s, some researchers have begun to investigate the modification of bipolar substrate to address the cell sealing and substrate corrosion. Meanwhile, inspired by this innovation of cell configuration, the BEs were subsequently introduced into the design of NMHBs, LIBs, and post‐LIBs. In 1985, the design principles of bipolar NMHBs were given in NASA technical memorandum.^[^ ^15^ ^]^ Subsequently from 1998 to 2007, the studies of bipolar NMHBs became a hot topic in the battery research, rendering numerous achievable technologies and published patents.^[^ ^16^ ^]^ In the same period, some alkaline batteries based on Zn and Al metals as the electrode materials were attempted to use BEs for high‐power operation.^[^ ^17^, ^18^, ^19^ ^]^ As for the LIB technologies using BEs, the earlier studies can be traced back to the Abraham et al.^[^ ^20^ ^]^ and Peled et al.^[^ ^21^ ^]^ in 1995. Later, Golodnitsky et al. contributed the basic understand and model design by using the Li/CPE/FeS_2_ batteries.^[^ ^22^ ^]^ In the last decade, the ever‐increasing enthusiasm of battery scientists have given impetus to the development of BEs in the fields of the LIBs and post‐LIBs. Especially in 2019, the BEs appeared in the post‐LIBs including lithium‐sulfur batteries (LSBs)^[^ ^23^ ^]^ and sodium‐ion batteries (NIBs).^[^ ^9^ ^]^ Figure 1d shows the timeline of these key works and development in the history of BEs.

### 2.1 Simplification in Battery Configuration with Reduced Weight, Size, and Cost

Rechargeable batteries are currently assembled by MEs, as shown in Figure 1b, where the electrode is coated with identical active material on both sides of the substrate. All electrodes are soaked in the shared electrolyte of the entire case. Each electrode is connected in parallel using an external wire. In contrast, BEs require the electrode to be separately coated with cathode and anode materials on each side, as shown in Figure 1c. The cathodic electrode of any cell unit is directly electronically connected with the anodic electrode of the next cell unit via a thin substrate. The electrolyte in each cell is isolated by a substrate and rubber rings, leading to switching off ionic transport in the electrolyte and averting the short circuit issue of the battery.^[^ ^24^ ^]^ All electrodes are connected in series without external accessories, such as taps and wires. There are only two taps at both ends of the battery stack to yield a high‐voltage output. Therefore, the maximum savings of inactive components in cell configuration is favorable to significantly reduce the weight, size, and cost of a battery, thus allowing for an enhanced specific/volumetric energy of a rechargeable battery.^[^ ^11^, ^12^, ^25^ ^]^

### 2.2 Enhancements in Battery Capacity/Power Densities and Thermal Management

From single cell to battery system, all these cells are integrated into a complex battery system with external circuits that are inactive materials to serve as auxiliary functions.^[^ ^26^ ^]^ Consequently, there is an estimated energy density loss of ≈40% in volume and ≈20% in weight. In the case of BEs, the bipolar batteries have a simplified cell configuration and shape because of no use of electric connectors and other accessories.^[^ ^11^ ^]^ The stacking thickness of all unit cells and the substrate area of a unit cell is used to calculate battery volume. The battery weight is close to the mass sum of all the components. To follow, the battery energy is known as the product of capacity and voltage. The capacity of bipolar battery is the same as that of a single unit cell, while the output voltage of bipolar battery is determined by the product of the number of unit cells in series and the voltage of each cell.^[^ ^10^ ^]^ Consequently, volumetric/gravimetric energy density of bipolar batteries is equal to battery energy divided by battery volume/energy, respectively. As expected, the rechargeable batteries using BEs have also a significant increase in volumetric/gravimetric energy density. Furthermore, the battery shape is readily tuned based on the application‐oriented design, resulting in maximized utilization of battery storage space in targeted devices. Therefore, these advantages of BEs are highly attractive for the design of rechargeable batteries employed in mobile electronics and electric vehicles.

Beyond the specific energy/volumetric benefits in a rechargeable battery, there is a significant enhancement of the specific power performance. Unlike the energy density, the power density of a battery is not restricted by thermodynamics,^[^ ^11^ ^]^ but mainly by the degree of polarization of the charging/discharging processes. In other words, for any rechargeable battery, there are no principled restrictions to high‐power operation. In contrast, the failure of high‐power operation is mainly attributed to the high polarization potential, rendering the working voltage beyond the cut‐off voltage. Regardless of the dynamic properties of electrode materials, the polarization potential of a rechargeable battery is determined by the current density and the internal resistance of cell construction, as shown in Equation (1).

(1) $$\begin{array}{ccc} \Delta U\; & = & (R_{i,\;\mathrm{electrolyte}}\;+R_{i,\;\mathrm{am}}+R_{i,\;\mathrm{separator}}+R_{i,\mathrm{am}/\mathrm{cc}}+R_{i,\;\mathrm{cc}\;}\\ & & +R_{i,\;\mathrm{cell}\;\mathrm{connector}})\;\times I \end{array}$$

where Δ*U*, *R*_i, electrolyte_ *, R*_i, am_ *, R*_i, separator_, *R*_i, am/cc_ *, R*_i, cc_ *, R*_i, cell connector_, and *I*, represent the polarization potential, resistance of electrolyte, active materials, separator, interface between active materials and a substrate, substrate, and cell connector and current density, respectively.

In the cell configurations of MEs and SEs, the working current essentially passes through the terminal tab of the electrode. Due to the small cross‐sectional area of the tabs (Figure 1a,b), high current density (*I*) formed in the tabs results in a serious voltage drop for decreasing battery capacity. Furthermore, once such a current density exceeds the security threshold, overheating and even fire accidents, will likely occur during the high‐rate charging/discharging processes.^[^ ^27^ ^]^ Therefore, corresponding approaches of using multiple tabs and cooling system are essentially required for high‐power rechargeable batteries. However, such an implementation in rechargeable batteries results in more complex electrode design and high‐cost manufacturing. The utilization of multiple tabs and cooling system also leads to the decrease in energy density of a battery. Moreover, the cells should be operated within their design specifications. The abuse of a battery over design specifications must be also taken into account due to strict requirements in battery safety.

Here, BEs inherently have the advantages to avoid the above cases. BEs enable rechargeable operation with no external cell connectors (Figure 1c). There is no value for *R*_i, cell connector_. Meanwhile, the electron flow is directly transferred through the substrate, which also has a negligible *R*_i, cc_ value. Furthermore, the electron flow is perpendicular to the substrate, where a large cross‐sectional area of the substrate significantly improves the current density and distribution. The low current density also endows low polarization and heat even at a high rate of operation where cooling system is no longer essential at all. Therefore, rechargeable batteries with a high rate of operation using BEs can perform without safety concerns.

### 2.3 Basic Requirements and Existing Disadvantages in Bipolar Electrodes

Despite various benefits in electrochemical performance, the success of BEs in rechargeable batteries critically depends on many requirements for the battery components. First, the substrates take part in the sealing process for the electrolyte. As such, the substrate is required to have excellent mechanical properties.^[^ ^10^ ^]^ Second, the electrolyte isolated in each cell must be very stable without decomposing gas. Batteries that swell to the detriment of electrolyte isolation must be controlled.^[^ ^28^ ^]^ Third, known that the cathode and anode are both coated on a substrate, this substrate must own excellent electrochemical stability at both high and low voltages. No chemical reactions of the substrate simultaneously occur in either the cathode or the anode. Fourth, each electrode unit has the same amount of electron transfer during the charging/discharging procedure. Overcharge and overdischarge of any unit cell is potentially induced by the inconsistency of electrode capacity, which is a special need of attention in BEs compared with SEs and MEs. Fifth, low cost in substrate materials and easy handling for good integration during production is highly desired in the industrial sector. Accordingly, compared with MEs and SEs easily assembled, the difficulty in achieving BEs makes it unattractive to some extent.

As well, there are some inherent disadvantages of the BEs to be clarified. First, as discussed prior, the BEs allow for a natural series structure without the additional external wires, compared with MEs and SEs. As such, the electrode inconsistency probably results in the accelerated decay of the other cells. The whole battery system becomes abortive once the failure of any unit occurred in this framework.^[^ ^29^ ^]^ By comparison, the damaged unit cells based on MEs and SEs only result in the capacity decay of the batteries. Therefore, the BEs are more fragile in battery performance than MEs and SEs. The larger the battery module, the higher battery replacement costs. Second, in real‐world conditions, a slight capacity loss has been observed with increasing series number of unit cells.^[^ ^30^ ^]^ It is unavoidable that the uneven electrode capacity results in a narrow range of voltage cut‐off to setup the overcharge and overdischarge protection. The achievable energy density of bipolar batteries may be only 80% of theoretical values. To this end, the battery management becomes more critical for diagnosing cell voltage and maintaining the health state of bipolar batteries.

## 3 Applications of the Bipolar Electrodes

### 3.1 Bipolar LABs

Before LIB technologies, LABs and NMHBs are well‐established energy storage systems that have mostly been used in the car startup, hybrid electric vehicle, and electric‐driven devices.^[^ ^8^ ^]^ Accordingly, they are the earliest users of BEs for improved specific energy/power. Their successful cases in roadmap of bipolar LABs in commercialization from lab to market and bipolar NMHBs in spacecraft applications are particularly worthy discussing.

#### 3.1.1 Lab Research of Bipolar LABs

Compared with traditional LABs, bipolar LABs have less mass/volume for improved energy densities in dimension and mass simultaneously. In detail, compared with the regular LABs, the bipolar LABs have ≈40% less volume or 60% the size as well as ≈30% less weight or 70% the mass. However, because of no suitable materials to seal diluted H_2_SO_4_ in individual cells, it has been difficult to achieve long‐lifespan bipolar LABs. Until the 1960s, the seals and vents were invented in bipolar LABs.^[^ ^14^ ^]^ Subsequently in 1990s, practically viable bipolar LABs were constantly reported. LaFollette et al.^[^ ^10^, ^31^ ^]^ offered basic fundamentals for the design, assembly, and production of bipolar LABs. Their contributions focus on the proposal of basic models and packaging technologies. Subsequently, challenges in bipolar LABs are mainly from their substrate essentially serving for the cathode and anode. Physical and chemical properties of bipolar substrates play a critical role in power performance, cycling lifespan, battery weight, and manufacturing cost.^[^ ^32^ ^]^ Especially, in the electrolyte of aqueous sulfuric acid, high corrosion stability, and high overpotential (H_2_–O_2_) are specially required.

To this end, considerable effort has been devoted to the research of substrate materials for bipolar LABs.^[^ ^12^, ^14^, ^33^, ^34^, ^35^ ^]^ For instance, Karami et al.^[^ ^33^ ^]^ reported using a lead‐tin alloy as the substrate material of bipolar LABs. Tin in the alloy is capable of increasing the hydrogen evolution overpotential, leading to a more stable interface for the sulfuric acid under strong oxidation conditions. Y. R. Lun‐Shu et al.^[^ ^35^ ^]^ first electroplated a layer of lead on a titanium substrate and subsequently performed a thermal treatment for an enhanced bonding effect between the lead and titanium. Lang et al.^[^ ^36^ ^]^ performed a series of research studies on the substrate of a bipolar LAB. The titanium foil was coated by pyrolytic carbon and TiO_2−_ *_x_* to enhance the corrosion resistance of the titanium metal in a H_2_SO_4_ electrolyte. These varied strategies and methods have been identified to be effective in enhancing the lifetime, illustrating the isolated necessity of inert layer between substrate and electrolyte.

**Figure 2:**
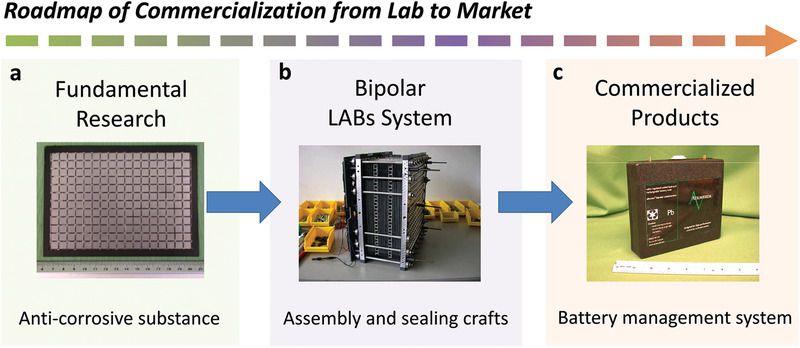
Roadmap of commercialization from lab to market: a) Planar view of an Ebonex plate for a bipolar LAB prior to pasting with active materials. Reproduced with permission.^[^ ^41^ ^]^ Copyright 2005, Elsevier. b) Photograph of the bipolar lead‐acid battery after assembly. Reproduced with permission.^[^ ^11^ ^]^ Copyright 2005, Elsevier. c) A valve regulated 12 V Ebonex bipolar lead‐acid battery. Reproduced with permission.^[^ ^39^ ^]^ Copyright 2004, Elsevier.

Besides essential surface modifications, several anti‐corrosive powders against the acid electrolyte have also been developed for bipolar substrates. Barium lead oxide (BaPbO_3_), which is a perovskite ceramic material with excellent conductivity, was reported by Paleska et al.^[^ ^37^ ^]^ as a substrate material. The surface of the BaPbO_3_ substrate was electroplated with a layer of lead by cyclic voltammetry. Such a BaPbO_3_ substrate has reduced weight without compromising the electrochemical properties. In another example, Reichman et al.^[^ ^38^ ^]^ dispersed several transition metal oxides, such as TiO_2−_ *_x_*, Mo_2_O_3−_ *_x_*, WO_3−_ *_x_*, Ni_2_O_5−_ *_x_*, and V_2_O_5−_ *_x_*, into polypropylene membranes, efficiently enhancing the conductivity of the polypropylene film. Such a composite film is very suitable for the substrate of bipolar LABs. Particularly, Ellis et al.^[^ ^39^ ^]^ developed a resin‐bonded composite as a bipolar substrate (**Figure** **2**a). TiO_2−_ *_x_* powder, fibers, and adhesives were mixed. The resultant slurry was strongly pressed by thermal pressure to achieve the fabrication of the substrate, which is readily scaled up in the industrial sector.

#### 3.1.2 Commercialization Process of Bipolar LABs

Along with the substrate development, some teams began to assemble package system. Saakes et al.^[^ ^11^, ^40^ ^]^ reported a series of achievements using a bipolar LAB system. Bipolar LABs can have a power density of 500 W kg^−1^, an energy density of 30 Wh kg^−1^, and a cycling life of 100 000 times, as the assistant power for hybrid vehicles at the startup/brake state. The working voltage of 80 V is readily enabled by stacking cell units (Figure 2b). In commercialization process, the industry community holds a great interest in bipolar LABs. The substrate proposed by Ellis et al.^[^ ^39^ ^]^ was subsequently developed by Atraverda Company, UK, and was registered with a trademark of Ebonex. The technology for preparing this substrate is also called Ebonex technology. Loyns et al.^[^ ^41^ ^]^ assembled bipolar LABs using an Ebonex substrate. The resultant bipolar LABs (Figure 2c) exhibited improved performance and lifetime.^[^ ^42^, ^43^ ^]^ In addition, the OPTIMA from Sweden and the VOLVO Automobile Company have jointly developed a bipolar LAB system, referred to as Effpower, for hybrid electric vehicles. The BPC Corporation of the United States has strongly invested the research of bipolar LIBs, consequently producing a 180 V, 60 Ah bipolar LAB system with a high specific power and a long cycle life. Such a practical demonstration confirms that bipolar LABs offer a high‐efficiency and low‐cost power assistant for hybrid vehicles.

### 3.2 Bipolar Alkaline Batteries (NMHBs and Al and Zn Batteries)

#### 3.2.1 Bipolar NMHBs

**Figure 3:**
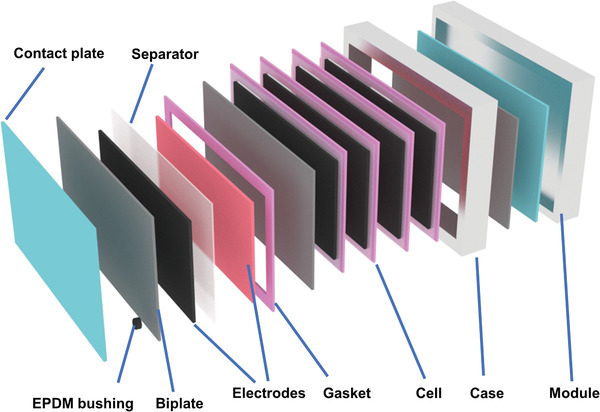
A high‐voltage module built from a bipolar Ni–MH cell arrangement.

As the case with bipolar LABs, the bipolar NMHBs with BEs allow for a 25% reduction in both weight and volume for higher energy and power capacities, compared with the regular ones with conventional electrode structure.^[^ ^44^ ^]^ Initially, Ohms's group^[^ ^45^ ^]^ reported a series of research studies including battery design, electrochemical performance, and heat management. Corresponding to the above principles, a bipolar NMHB case of 1000 W kg^−1^ was assembled using BEs. Both a working voltage of >50 V and a long lifespan of >1000 cycles were readily achieved. Similarly, Yartys et al. constructed a 10‐cell bipolar NMHB with the output voltage of 12.5 V.^[^ ^46^ ^]^ Such an assembled configuration of high‐voltage cell is schematically shown in **Figure** **3**. Subsequently, Deng and co‐workers^[^ ^47^, ^48^, ^49^ ^]^ performed the variety of improvements to advance BEs for NMHBs. For example, a gas chamber was added to a bipolar NMHB.^[^ ^48^ ^]^ The gas phases were interconnected via these gas chambers, but the electrode electrochemistry was still independent. Such a structural design helps transfer the reaction gases between the subcells and balances the interpressure of the subcells. In addition, a nanometer copper oxide as an anodic additive was also reported by Deng et al.^[^ ^47^, ^48^ ^]^ to enhance electrochemical performance of bipolar NMHBs. The addition of nanometer copper oxide in the anode efficiently reduces the contact resistance and charge transfer resistance.

Beyond academic research, the bipolar NMHBs have been massively explored well in spacecraft applications.^[^ ^16^, ^50^ ^]^ The NASA department devoted much effort into bipolar NMHBs. Based on a NASA technical memorandum,^[^ ^15^, ^51^ ^]^ some design principles including oxygen management, electrolyte management and reservoiring, thermal management, and component growth management have been summarized for bipolar NMHB cells. These principles can guide how to improve cell performance and cycle lifetime.

#### 3.2.2 Bipolar Al and Zn Batteries

Available and inexpensive metals, such as Zn and Al, with multi‐electron redox are a promising candidate for energy storage system.^[^ ^52^, ^53^ ^]^ These batteries also have the record of using BEs.^[^ ^17^, ^18^, ^19^ ^]^ For instance, Rota et al. successfully assembled bipolar Al‐O_2_ batteries.^[^ ^18^ ^]^ Similarly, Muller and coworkers developed a 100 W bipolar Zn‐O_2_ batteries.^[^ ^17^ ^]^ After a stacking design including seven cells, the battery stack can deliver the peak voltage output of 10 V. In addition, Ghaemi et al.^[^ ^54^ ^]^ proposed a series of improvements in bipolar cells, such as Optalloy treated zinc anodes and an artificial neural network, to address gas evolution issues. Recently, Ahmed et al.^[^ ^19^ ^]^ developed high‐current bipolar Zn batteries where Zn is directly used as active materials and bipolar substrate. The discharge current capability of 500 mA cm^−2^ with three cells was achieved. These attempts have demonstrated the flexibility of metal batteries using BEs in alkaline electrolyte.

### 3.3 Bipolar LIBs

Compared with LABs and NMHBs with aqueous electrolytes, LIBs with organic electrolytes inherently process a higher voltage output in each cell.^[^ ^4^ ^]^ When further combined with BEs, bipolar LIBs will be more competitive in the application of electric and hybrid electric vehicles while these electrified transportations need for the battery packs with working voltage of 300–500 V.^[^ ^55^ ^]^ Thus those high voltage battery packs require several hundred of cells to be connected in series. The role of BEs is critical for less using wires for cell series connection and maximally simplifying this process. Moreover, such a combined high‐voltage and high‐current delivery also enables fast acceleration and charging behaviors.

**Figure 4:**
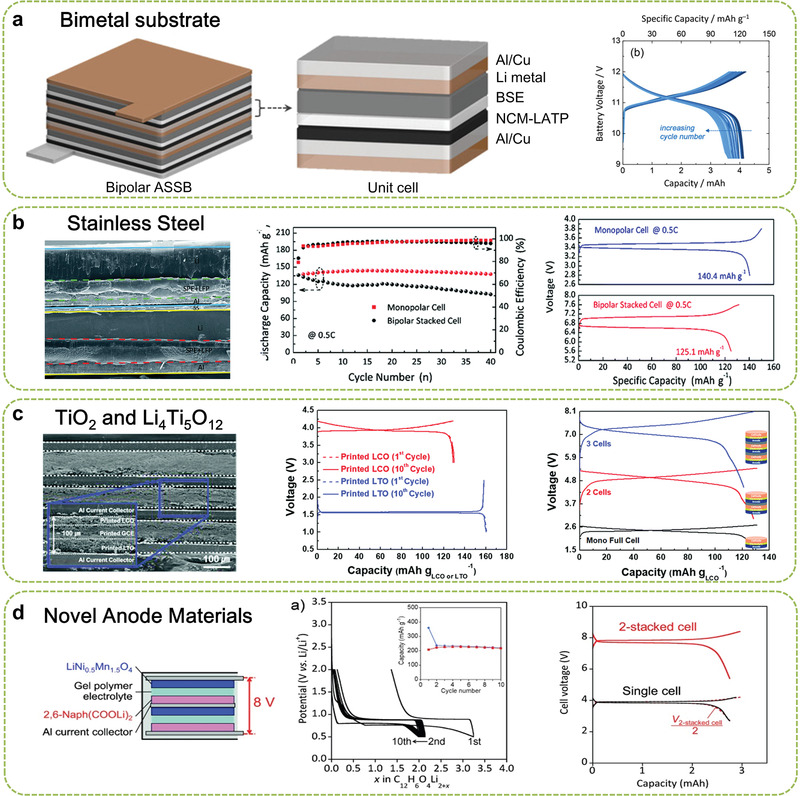
Typical strategies for enabling BEs in LIBs: a) Schematic illustration of the bipolar ASSBs and galvanostatic charge–discharge profiles of three‐cell bipolar cells. Reproduced with permission.^[^ ^56^ ^]^ Copyright 2018, Wiley‐VCH. b) SEM image of inner structure of bipolar LIB using SS and comparison of charge/discharge profiles after the fifth cycle. Reproduced with permission.^[^ ^59^ ^]^ Copyright 2018, Royal Society of Chemistry. c) Schematic of inner structure of bipolar LIB, wherein both LiCoO_2_ cathode and Li_4_Ti_5_O_12_ anode use the Al foil as a current collector, as well as the preparation processing. Reproduced with permission.^[^ ^28^ ^]^ Copyright 2018, Royal Society of Chemistry. d) Charge/discharge profiles for an Li/2,6‐Naph(COOLi)_2_ cell (inset: corresponding cycling performance) and charge/discharge profiles of two stacked bipolar 2,6‐Naph(COOLi)_2_/LiNi_0.5_Mn_1.5_O_4_ cells (inset: corresponding cell configuration). Reproduced with permission.^[^ ^64^ ^]^ Copyright 2014, Wiley‐VCH.

Lee et al. assembled multilayered, bipolar, all‐solid‐state LIBs by using a novel solid electrolyte.^[^ ^56^ ^]^ The cathode and anode materials are coated on one side of Al and Cu foils, respectively, as shown in **Figure** **4**a. The voltage range from 9.2 to 12.0 V has been obtained by using a three‐cell bipolar LIB. Regrettably, the energy density of this resultant battery is to some extent impacted by the use of bimetal substrate of Al and Cu foils. It is attributed to the fact that the LIB current collectors use only Al and Cu foils for the cathode and the anode,^[^ ^57^, ^58^ ^]^ respectively. The side reactions include the Li‐alloying of Al foils at low potentials (≈0.3 V vs Li/Li^+^)^[^ ^57^ ^]^ and the oxidization of Cu foils at high potentials (>3.5 V).^[^ ^58^ ^]^

To this end, an alternative stainless steel (SS) is used as the substrate for bipolar LIBs, which was well exemplified by the works of Abraham et al.^[^ ^20^ ^]^ and Livshits et al.^[^ ^22^ ^]^ Recently, Chen et al.^[^ ^59^ ^]^ used the SS substrate to assemble solid‐state bipolar LIBs (Figure 4b). Such a two‐unit bipolar LIB based on LiFePO_4_ cathode and Li anode successfully delivers a high voltage of 6.07 V. Meanwhile, a further evidence of the SS substrate used in LIBs is provided by Honma et al., who developed a polymer electrolyte matched with the BEs to achieve bipolar LIBs.^[^ ^30^ ^]^ These high voltage plateaus of 6.7 and 10.0 V are readily achieved by using two and three stacking of single units, respectively. After 200 cycles, the reversible capacity and CE value are effectively maintained, be indicative of high chemical and electrochemical stabilities of SS substrate for the cathode and anode. Kasnatscheew et al. further analyzed the origins of an immediate short‐circuit in bipolar LIBs using sulfide‐based polymer electrolyte, and emphasized the area‐oversized SS substrates in place of Cu ones.^[^ ^29^ ^]^

Besides using inert substrates, the approach of using high‐voltage anode materials, such as TiO_2_ and Li_4_Ti_5_O_12_, also is viable to avoid the Al–Li alloying reaction and thus achieve bipolar LIBs. The Al foils are designed for both cathode and anode. For example, Sato et al.^[^ ^60^ ^]^ and Yoshima et al.^[^ ^61^, ^62^ ^]^ have successfully achieved a high working voltage of 12 V by assembling five stacking of bipolar LIBs. Such an assembled cell also shows excellent cycle durability and rate operation up to 10 C. In addition, Lee et al. reported a series of bipolar LIBs.^[^ ^28^, ^63^ ^]^ A flexible, shape‐versatile, all‐solid‐state bipolar LIB was reported for the applications in wearable electronic devices (Figure 4c).^[^ ^28^ ^]^ As the cell number increased, the working voltages of the resulting cells increased from 2.4 V (mono cell) to 5.4 V (two cells) to 7.2 V (three cells). Despite the success of above route to bipolar LIBs, a significant voltage reduction of unit cell results in a serious loss in energy density, making it unacceptable in practical view.

In pursuit of high‐energy‐density LIBs, the need for reducing the anode voltage platform strongly encourages innovations in the battery materials. Ogihara et al.^[^ ^64^, ^65^ ^]^ synthesized an organic anode material of 2,6‐Naph(COOLi)_2_ with a discharge platform of 0.8 V, which offers a sufficient buffer to avoid the lithiated reaction of Al foils and allows a more safe discharging operation. Thus, the output voltage of 8 V was easily achieved by two units based on BEs, as shown in Figure 4d. This route has opened up a new avenue for organic electrode materials employed in LIB.

### 3.4 Post‐LIB Battery Technologies (Li‐S Batteries and Na‐Ion Batteries)

**Figure 5:**
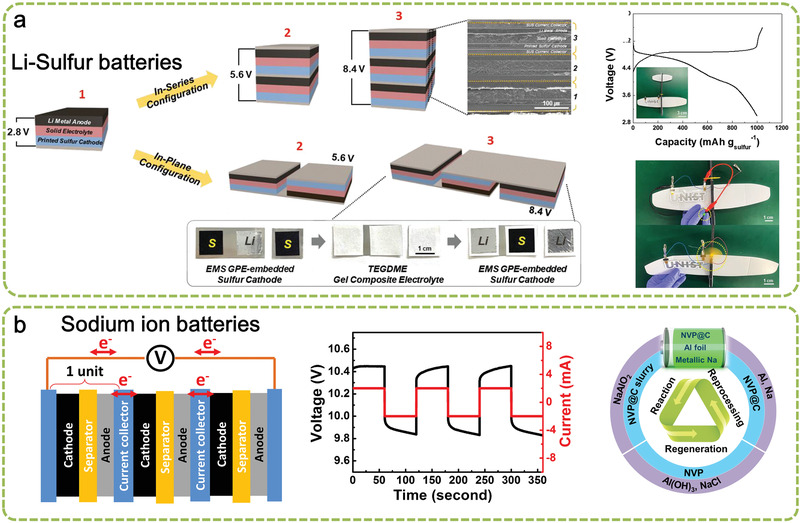
a) Schematic representation depicting the fabrication procedure of the printed bipolar ASSLSB, along with the SEM image (in‐series configuration) and photographs (in‐plane configurations), charge/discharge profiles (0.1 C) of the bipolar (2 cells connected in‐series) ASSLSB directly fabricated on the curvilinear surface of a toy aircraft, and photographs showing the operation (LED lamp and propeller of the toy craft) of the printed bipolar ASSLSB. Reproduced with permission.^[^ ^23^ ^]^ Copyright 2019, Wiley‐VCH. b) A three‐unit bipolar NIB cell including a schematic of the electrode structure in a battery pack, high current/voltage curves in the charge and discharge process at a 10 C rate for 60s and schematic close‐loop utilization of a proposed NIB unit. Reproduced under the terms of the CC‐BY Creative Commons Attribution 4.0 International license (https://creativecommons.org/licenses/by/4.0).^[^ ^9^ ^]^ Copyright 2019, The Authors, published by Springer Nature.

Next‐generation energy storage technologies are frequently emphasized as high‐energy‐density and low manufacturing cost.^[^ ^66^ ^]^ As the most promising candidate for high‐capacity Li‐storage, the LSB is an appealing technology. Lee et al. further developed the all‐solid‐state bipolar LSBs.^[^ ^23^ ^]^ The SS is still used as the substance for Li metal and printed sulfur cathode. Bipolar LSBs are also assembled by two types of BEs with in series configuration (face‐to‐face) and in plane configuration (side‐by‐side). The latter can be described as semi‐polar structure, where positive and negative electrodes are placed on the edge of the shared current collector, as shown in **Figure** **5**a. Such a design offers an appealing advantage that the battery with a flat and flexible shape achieves high voltage output, rendering a flexible high voltage battery and be more compatible in targeted applications than the former. Notably, this side‐by‐side type of BEs, although which has relatively higher internal resistance compared with face‐to‐face, still remains much lower in internal resistance than the conversional connection of MEs and SEs.

NIBs are considered a viable alternative to LIBs in the application of large‐scale energy storage.^[^ ^67^ ^]^ Na certainly has no alloy reaction with Al, providing a medium as the substrate for BEs.^[^ ^9^ ^]^ As such, Al foils instead of Cu foils can be used for the NIB anode. Furthermore, Al has a material density and cost advantage over Cu, allowing a lower manufacturing cost and improved energy density. Liu and coworkers, for the first time, proposed a recyclable concept for high‐performance NIB enabled by adopting BEs (Figure 5b).^[^ ^9^ ^]^ Given the feature of BEs for fast electron transfer, NIBs can exhibit exceptional electrochemical performance in terms of high‐power correspondence. Notably, the simplification of metallic current collector in NIBs also contributes to recycling ≈100% Na_3_V_2_(PO_4_)_3_ and ≈99.1% elemental Al without the release of toxic wastes, resulting in a solid‐component recycling efficiency of >98.0%.

## 4 Conclusion, Challenge, and Outlook

**Figure 6:**
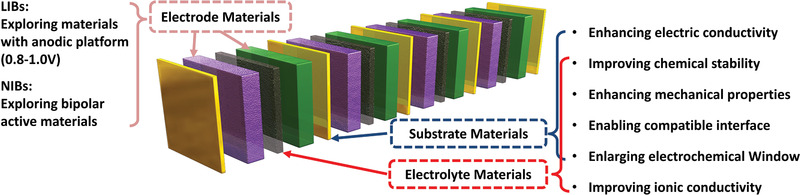
Summary of strategies toward bipolar cell configuration, including electrode materials, substrate materials, electrolyte materials.

As reviewed in the article, BEs have obvious advantages in simplifying battery components and improving power performance, as summarized in each section. In particular, several commercially viable cases of LABs and NMHBs using BEs demonstrate that these technological barriers in bipolar battery manufacturing can be overcome. However, state‐of‐the‐art LIBs and post‐LIB batteries, such as LSBs and NIBs, have few cases in which to use BEs. On the one hand, the lack of various suitable electrode materials, electrolyte materials, and substrate materials is a critical reason for BEs. For instance, the substrates not only serve as the current collector for both cathode and anode, but also contribute to individually seal the electrolyte compartment of each cell in the series. These requirements are rarely mentioned in LIBs. On the other hand, a reality of BEs is needed for corresponding battery management system to monitor and balance on the series. Fortunately, the former is potentially addressed by the development of solid‐state electrolyte (**Figure** **6**).^[^ ^66^, ^68^ ^]^ The latter has the experience shared from the commercial success of bipolar LABs and NMHBs. By comparison, the advances in electrode materials, substrate materials, and electrolyte materials need for substantial effort to be made. Accordingly, new opportunities may be created upon navigating through these challenges.

### 4.1 Substrate Materials

The compatibility of current collectors in cathode and anode is critical for BEs. Zn or Al have been reported as both active material and current collector, allowing an anode integration in bipolar batteries. The feasibility of current collectors for the cathode and anode strongly contributes to the BE adoption in rechargeable batteries. As the case of LIBs, bimetal current collector based on Cu and Al, which are isolated from each other, can enable the success of the BEs in battery system. This sacrifice is to use more metal current collector, leading to reduced energy density and increased batter cost. Currently, the industrial thickness of Cu and Al foils is 8–12 µm. The thickness threshold of blocking Li ion diffusion without crossing the self‐body lacks of relevant investigations. In addition, the SS as an alternative current collector has been reported for the cathode and anode, demonstrating its excellent compatibility with the cathode and anode.^[^ ^20^, ^22^, ^52^, ^59^ ^]^ Problems in bipolar stacked configuration involved with the stiffness and thickness of the SS substrate, but the detailed investigations of SS for BEs are still needed. The low thickness of SS substrate is favorable for increasing energy density and reducing battery volume. As for post‐LIBs, Al foils used for both cathode and anode advantageously enable bipolar NIBs with simplified cell configuration. When Al foils are used as the shared current collector in bipolar NIBs, its features of low material density and cost are advantageous compared to Cu foils.

### 4.2 Electrode Materials

Regardless of the impact by current collectors, electroactive materials with anodic platform range from 0.8 to 1.0 V for LIBs is highly desired, not only enabling no Al/Li alloying reactions, but also affording relatively high output in paired with the cathode. To this end, organic materials have hold a great promise to satisfy above strict requirements. In addition, these ultra‐high rate battery technologies and materials^[^ ^69^ ^]^ are worthy of attention, as the BEs are very compatible for varied battery technologies. In this context, electroactive materials have high‐rate capability to match with high‐power operation of BEs, critically requiring the electroactive materials with an open crystal framework, high specific surface area, and rich porous structure allow for the high dynamics of Li insertion/desertion reactions and fast ionic diffusion.^[^ ^70^ ^]^ Relatively high electric conductivity, which is enabled by the modification of metal or carbon coating, effectively relieves the cell polarization under high‐rate operation. What is more, electrode materials that have double redox couples,^[^ ^71^ ^]^ such as Na_0.8_Ni_0.4_Ti_0.6_O_2_, Na_0.6_C_r0.6_Ti_0.4_O_2_, and Na_0.9_Ni_0.45_Ti_0.55_O_2_, should be preferentially developed in the system of NIBs. These could be used for both cathode and anode to assemble symmetric cells.^[^ ^72^ ^]^ Such a symmetric cell structure is readily transferred to bipolar cell configuration.

### 4.3 Electrolyte Materials

Currently, there is substantial interest in developing novel solid‐state electrolytes for solid batteries.^[^ ^73^ ^]^ Considering the integration of BE in solid‐state LIBs, incremental exploration and optimization of solid‐state electrolytes would be toward fast‐ion conductors, such as Li_10_GeP_2_S_12_ and Li_7_P_3_S_11_, because solid‐state electrolytes with low conductivity seriously diminished the benefits of BE in specific power.^[^ ^74^ ^]^ Moreover, an affiliated challenge is to stabilize the rate‐oriented interface between electrolyte and electrode. Operation of high rate charging or discharging is more susceptible to lithium dendrite, serious polarization, and decomposed by‐products.^[^ ^7^ ^]^ Therefore, how to achieve an intimate physical contact and excellent chemical/electrochemical compatibility between electrode and electrolyte should be carefully considered. More comprehensive understanding of the intrinsic mechanisms enabled by advanced detection techniques is of great important significance. For example, cryo‐electron microscopy to probe electron‐beam‐sensitive interface between electrode and electrolyte may become insightful.

### 4.4 Engineering Technologies

The manufacturing issues of the BEs employed in rechargeable batteries are very worthy of attention. Exceptionally high requirements in electrode consistency in term of capacity, voltage, and performance decay really challenge the current technologies in electrode fabrication and performance diagnosis. What is more, among substrate, electrode, and electrolyte, their interfacial compatibilities are first evaluated to ensure faultless uniformity in each unit. Overlooking the mismatch of each battery component brings in the serious consequences, such as overcharge, overdischarge, and swell. If the activation of unit cells is essentially designed for voltage modulation and capacity screening, the manufacturing of bipolar batteries has high risk in cost. While activation‐free bipolar batteries bring in highly strict requirements in the synthesis of electroactive materials and fabrication of electrode.

#### 4.4.1 Prospects of the Bipolar Electrodes

In addition to new impetus from materials, emerging concepts, such as wearable technology applications, solid‐state electrolyte LIBs, fast spraying fabrication, and recyclable rechargeable batteries, also offer an initiative to advance BEs into LIBs and post‐LIB batteries. Such an exciting prospect is impending. Therefore, at the end of this review, we provide several benefits to be expected as follows.

**Figure 7:**
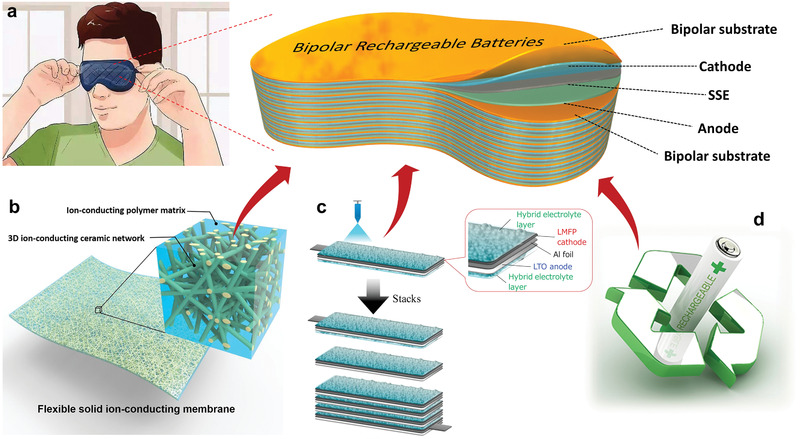
Schematic illustration of BEs in LIBs and NIBs combined with emerging concepts. a) Wearable applications. b) Solid‐state electrolyte. c) Fast spraying fabrication. d) Recyclable design. b) Reproduced with permission.^[^ ^82^ ^]^ Copyright 2016, The Authors, published by National Academy of Sciences. c) Reproduced with permission.^[^ ^62^ ^]^ Copyright 2016, Elsevier.

First, BEs are capable of enabling shape‐versatile design of LIBs. In general, our understanding of wearable technology applications demands flexible LIBs. Namely, a flexible LIB can offer a degree of flexibility without losing the energy supply.^[^ ^75^ ^]^ But more importantly, wearable technology applications must have irregular shapes to fit the human body, thus requiring a shape‐versatile design of LIBs.^[^ ^28^ ^]^ Traditional square and circular batteries are far from satisfactory. In contrast, bipolar cells built by in‐series or in‐plane configurations exhibit exceptional flexibility in battery shape, as shown in **Figure** **7**a. In addition, high safety of the battery requires abuse tolerance at high‐rate operation, which is highly preferred in BEs for wearable devices.

Second, BEs can excellently match with the solid‐state electrolyte. Their combination strongly promises low cost, high specific, and high energy of solid‐state batteries. Specifically speaking, solid‐state electrolyte is free of the sealing process compared with liquid electrolyte,^[^ ^76^ ^]^ resulting in a simple and compact battery configuration (Figure 6) and thereby contributing to low cost manufacturing and high specific energy of rechargeable batteries. In addition, the solid‐state electrolytes are readily processed into the various shapes (Figure 7b). It has been above mentioned that high rate charging or discharging are more susceptible to overheat. Non inflammable advantage of solid‐state electrolyte further enhances thermal abuse resistance.^[^ ^77^ ^]^

Third, BEs possibly reshape the electrode manufacturing process instead of routine practices involving solid‐state battery. Recently, akin to such a technology, Yoshima et al.^[^ ^61^, ^62^ ^]^ described that the all‐solid‐state electrolyte of Li_7_La_3_Zr_2_O_12_ was sprayed to construct a layer‐by‐layer 12 V‐class bipolar battery, as shown in Figure 7c. Such all‐solid‐state bipolar LIBs exhibited a simple fabrication and wide temperature operation as well as maximally achieved an energy density of 90 Wh kg^−1^ and an output power density of 1500 W. More cases that relevant to 3D printed LIBs have already demonstrated this viable route in practical cell setting.^[^ ^78^ ^]^ Moreover, this advantage of fast fabrication is more brilliant by the effective integration in shape‐versatile battery.

Fourth, BEs make the battery simple and green. Currently, waste batteries have recently raised environmental concerns due to their toxic materials.^[^ ^79^ ^]^ Indeed, recycling is an ideal solution, not only addressing environmental concerns induced by waste materials, but also alleviating the shortage of critical materials through osed‐loop cycling.^[^ ^80^ ^]^ However, the initial design of LIBs does not consider the recovery process. High cost of manually disassembling waste LIBs makes the recycling process unprofitable.^[^ ^81^ ^]^ In this context, pyro‐ and hydrometallurgical methods to directly dispose of scalable waste LIBs are often adopted in industrial community, but require high energetic cost and massive chemical agents, leading to a subsequent environmental threaten. In contrast, if the initial design of LIBs using BEs can reduce the species of battery components, the efficient separation of spent LIBs and the direct regeneration/reuse of electrode materials are possible, which has been exemplified by the bipolar NIBs.^[^ ^9^ ^]^

## Conflict of Interest

The authors declare no conflict of interest.
